# GLP-1 receptor agonist–induced diabetic ketoacidosis: A case report

**DOI:** 10.1097/MD.0000000000039799

**Published:** 2024-09-27

**Authors:** Jiaming Zhang, Ying Ma, Qianhe Zu, Xiaohui Wang, Yao Zhang

**Affiliations:** a Department of Endocrinology and; b Office of Academic Research, Affiliated Hospital of Hebei University, Baoding, China; c Hebei University School of Basic Medical Sciences, Baoding, China.

**Keywords:** 1RAs, adverse drug reactions, diabetic ketoacidosis, GLP, LADA

## Abstract

**Rationale:**

Glucagon-like peptide-1 is an endogenous incretin that plays an active role in weight loss and hypoglycemia. Dulaglutide is a long-acting glucagon-like peptide-1 receptor agonist (GLP-1RA), which has been approved for the treatment of patients with type 2 diabetes (T2D). GLP-1RAs can increase insulin secretion and inhibit glucagon release, thereby leading to a decrease in blood glucose levels within the body. Specifically, GLP-1RAs control postprandial blood glucose levels by inhibiting hepatic glucose production and delaying gastric emptying. However, attention should be given to gastrointestinal adverse reactions. There are currently a few cases of GLP-1RA causing diabetic ketoacidosis (DKA).

**Patient concerns:**

The following report details the case of a 50-year-old Chinese female who has been living with diabetes for 12 years. Initially diagnosed with T2D, she was subsequently identified as a patient with latent autoimmune diabetes in adults (LADA) following treatment. The patient presented severe nausea, vomiting, and fatigue 1 day after injecting dulaglutide 1 time and discontinuing insulin therapy. She was diagnosed with severe DKA in the emergency department.

**Diagnoses:**

LADA and DKA.

**Interventions:**

Changed from dulaglutide to insulin therapy.

**Outcomes:**

After discontinuing dulaglutide and switching to insulin for blood glucose reduction, the patient’s DKA was corrected, and blood glucose levels returned to normal.

**Lessons:**

This case suggests that clinicians should be alert to patients with severe DKA in cases of severe gastrointestinal adverse reactions after the use of GLP-1RAs. In addition, in most countries, GLP-1RAs are administered to patients with T2D, but we should consider the use of GLP-1RAs in patients with type 1 diabetes and LADA.

## 1. Introduction

Glucagon-like peptide-1 (GLP-1) receptor agonists (GLP-1RAs) are effective in the treatment of type 2 diabetes (T2D).^[1,2]^ GLP-1RAs act as hypoglycemic agents while promoting weight loss through their effects on appetite and gastric emptying^[3,4]^ and play a role in lowering glycated hemoglobin (HbA1c).^[5]^ Dulaglutide is a subcutaneous GLP-1RA approved for monotherapy or as an add-on to other antihyperglycemic agents, including oral antihyperglycemic agents and insulin, in adults with T2D. At present, GLP-1RAs have not been approved for the treatment of type 1 diabetes. Despite this, some clinical studies are exploring the combination of GLP-1RA and insulin to seek more effective methods of blood glucose control. The results of these studies may have certain reference values for patients with type 1 diabetes. However, GLP-1RAs are also associated with adverse reactions, the most common being gastrointestinal reactions such as nausea, vomiting, and diarrhea.^[6]^ Due to the adverse effects of these drugs, we should pay attention to the emergence of diabetic ketoacidosis (DKA) in patients with type 1 diabetes and latent autoimmune diabetes in adults (LADA) during treatment.

## 2. Case presentation

A 50-year-old female with a background of diabetes was diagnosed in 2009, and her body mass index (BMI) was 24.46 kg/m^2^. She was given oral hypoglycemic drugs for 14 months after her diagnosis. After 14 months of oral antihyperglycemic agent treatment at the beginning of the disease, her blood glucose control was substandard. Patients with recurrent DKA, poor β-cell function, glutamic acid decarboxylase antibodies, and insulin autoantibody positivity were diagnosed with LADA in 2011. She switched to regular insulin injections, and her blood glucose fluctuated greatly. She also had disorders of lipid metabolism and hyperuricemia; her total cholesterol was 6.49 mmol/L, her triglyceride was 1.06 mmol/L, her low-density lipoprotein cholesterol was 3.65 mmol/L, and her uric acid was 629.94 µmol/L. The patient stopped using insulin to instead use dulaglutide 6 days ago. Soon after the first injection of dulaglutide, the patient experienced severe nausea, vomiting, fatigue, and loss of appetite. Three days later, the patient visited the emergency department due to severe nausea and vomiting. Laboratory tests revealed a random blood glucose level of 9.85 mmol/L, and blood gas analysis showed a pH of 7.34. The patient received targeted treatment and was discharged after their nausea and vomiting symptoms improved. However, the patient again experienced vomiting and dyspnea after leaving the hospital, and these symptoms lasted for 2 days before the patient presented to the emergency department again. The arterial blood gases indicated a significant metabolic acidosis (pH, 7.02; O_2_, 14 mm Hg; BE, −25.5; anion gap, 36 mmol/L; and lactate, 2.8 mmol/L). Her blood glucose level was 27.85 mmol/L. Afterward, the patient was transferred from the emergency department to the endocrinology ward. Laboratory tests revealed the following blood gas analysis results: pH, 7.02; extracellular fluid alkali reserve, −27.4 mmol/L; residual alkali, −25.5 mmol/L; and glucose, 26.7 mmol/L. Blood ketone body positive and 10.6% HbA1c. The diagnosis was DKA. She was treated with restored intravascular volume to correct tissue hypoperfusion and low-dose insulin treatment after discontinuation of dulaglutide. On the second day, the patient’s fasting glucose level was 7.23 mmol/L: blood gas, pH 7.35; extracellular liquid alkali reserve, −7.4 mmol/L; residual alkali, −6.6 mmol/L; actual bicarbonate, 18.2 mmol/L; and standard bicarbonate, 19.8 mmol/L. She continued to receive fluid rehydration and insulin therapy. Reexamination on the third day showed that there were no obvious abnormalities in blood gas analysis, DKA had been corrected, and blood glucose control had reached the standard. Finally, we developed a plan for intensive insulin therapy for the patient.

## 3. Discussion

DKA is a serious complication that occurs when diabetes mellitus is not properly controlled, and it is linked to higher rates of morbidity and mortality. Despite progress in DKA diagnosis and management, it remains a leading cause of hospitalization and death in children and adults, particularly in developing countries. DKA is a leading cause of death for children and adolescents with type 1 diabetes. It causes about 50% of the deaths of patients with diabetes under the age of 24 years. In previous cognition, DKA was thought to rarely occur in patients with T2D, and it was usually a specific clinical manifestation in patients with type 1 diabetes. However, in recent years, it has been found that about one-third of patients diagnosed with DKA are confirmed to have T2D. DKA is usually the first clinical manifestation in patients with type 1 diabetes,^[7]^ but DKA is rarely observed in patients with LADA.^[8,9]^ Patients with LADA are often misdiagnosed with T2D, but DKA is more prevalent and difficult to improve in patients with LADA than in patients with T2D.

GLP-1RAs are a class of incretin-based therapies for the management of hyperglycemia and are also among the most effective drugs for the treatment of obesity.^[3,4,10,11]^ These agents interact with various physiological mechanisms involved in T2D by boosting insulin production and curbing glucagon to regulate blood glucose levels. In addition, they temporarily decelerate gastric emptying, diminish appetite, and promote weight loss and other metabolic enhancements.^[12–14]^ GLP-1RAs are currently approved by the US Food and Drug Administration and the European Medicines Agency for the treatment of patients with T2D,^[15]^ and there is considerable evidence that they are also effective in the treatment of obesity and type 1 diabetes.^[3,4,10,11,16,17]^ However, there are few studies and reports on GLP-1RAs for the treatment of type 1 diabetes–induced DKA. In a study of 835 patients with type 1 diabetes who were enrolled in the study, the inclusion criteria are given as follows: the duration of type 1 diabetes was at least 1 year, the age was at least 18 years old, BMI was ≥20 kg/m^2^, they had received multiple daily insulin injections or continuous subcutaneous insulin infusion for at least 6 months, and the level of glycosylated hemoglobin was between 7.0% and 10.0% (53.0–85.8 mmol/mol). The insulin dose should be stable for at least 3 months. During the 26-week trial, only 47 of these 835 liraglutide users reported ketosis, and 37 of them reported symptomatic ketosis.^[16]^ In another study, 1398 patients with type 1 diabetes were enrolled to receive liraglutide, and the inclusion criteria for the population are given as follows: screening candidates with type 1 diabetes diagnosed clinically within the previous 12 months, receiving either basal insulin or continuous subcutaneous insulin infusion treatment for at least 6 months, with stable insulin therapy for the past 3 months, BMI ≥20 kg/m^2^, and aged between 18 and 75 years. A wide range of HbA1c levels (7.0%–10% [53–86 mmol/mol]) were designated as inclusive of patients with type 1 diabetes to ensure clinical representation of the trial population. Only 8 ketoacidosis events were reported during the 52-week trial.^[17]^ The above 2 studies demonstrate that GLP-1RA is effective in treating type 1 diabetes by stimulating insulin secretion and biosynthesis that are glucose-dependent, inhibiting glucagon secretion and gastric emptying, and reducing food intake. However, studies on the application of GLP-1RAs in patients with LADA are rare, and there is a lack of evidence-based medical evidence on whether GLP-1RAs are beneficial. In summary, GLP-1RAs have positive effects on glycemic control, promoting weight loss, reducing HbA1c levels, and reducing insulin dosage; however, their use is also accompanied by adverse reactions, especially gastrointestinal adverse reactions. Studies have shown that dulaglutide causes the lowest number of intolerable gastrointestinal adverse reactions, while liraglutide and semaglutide have the highest.^[18]^ Although these adverse reactions are very similar to DKA, there is no evidence that DKA is an adverse reaction associated with these GLP-1RAs. However, there are individual reports of DKA/euglycemic DKA after flat insulin withdrawal and dulaglutide application in patients with T2D.^[19,20]^ This is similar to what happened in our case. Two of the above patients were treated with only dulaglutide while completely stopping insulin and medication, followed by polydipsia, polyuria, nausea, and vomiting. Because of these triggers, DKA follows. The patient in this case was a patient with LADA whose islet function nearly completely failed. Considering that the application of GLP-1RAs could reduce the patient’s blood glucose and weight, it could not replace the use of insulin completely to avoid severe DKA and other acute complications. However, although there are cases of DKA/euglycemic DKA, we cannot determine whether these cases were accidental. At present, the evidence in the literature is still limited, and additional studies are needed to confirm whether DKA is directly related to the application of GLP-1RAs. We also considered whether the efficacy and adverse reactions of GLP-1RA administration were related to the BMI of the selected population. Through a literature search, we found that the median BMI of the selected population was 29 kg/m^2^,^[16,17]^ while the BMI of these patients was only 24 kg/m^2^. Considering whether BMI is related to the effects of GLP-1RAs and whether the BMI of the population is large, patients with a greater BMI are more likely to benefit from GLP-1RA treatment and less likely to experience DKA.

## 4. Conclusion

At present, GLP-1RAs are widely used in people with T2D who are overweight, and these beneficial effects are indeed significant for most patients with T2D; therefore, GLP-1RAs have become increasingly important in the treatment of diabetes in recent years. However, a small number of patients with T2D have reported conditions similar to DKA although we have not found any case reports of severe ketoacidosis in patients with LADA after the application of GLP-1RA through literature retrieval at present. However, for patients with type 1 diabetes and patients with LADA who are prone to ketoacidosis, we need to pay more attention to the gastrointestinal reactions when applying GLP-1RA. In addition, among the patients with diagnosed T2D, a small proportion of them are patients with undiagnosed LADA. Thus, attention should be given to evaluating islet function in patients with T2D treated with GLP-1RAs. If patients have poor islet function, caution should also be taken when administering GLP-1RAs.

## Author contributions

**Conceptualization:** Jiaming Zhang, Ying Ma, Yao Zhang.

**Methodology:** Jiaming Zhang, Yao Zhang.

**Writing – original draft:** Jiaming Zhang, Xiaohui Wang, Yao Zhang.

**Writing – review & editing:** Jiaming Zhang, Qianhe Zu, Yao Zhang.

**Supervision:** Yao Zhang.

## References

[1] Nauck M. Incretin therapies: highlighting common features and differences in the modes of action of glucagon-like peptide-1 receptor agonists and dipeptidyl peptidase-4 inhibitors. Diabetes Obes Metab. 2016;18:203–16. 26489970 10.1111/dom.12591 PMC4785614

[2] Aroda VR. A review of GLP-1 receptor agonists: evolution and advancement, through the lens of randomised controlled trials. Diabetes Obes Metab. 2018;20(Suppl 1):22–33. 29364586 10.1111/dom.13162

[3] Cornell S. A review of GLP-1 receptor agonists in type 2 diabetes: a focus on the mechanism of action of once-weekly agents. J Clin Pharm Ther. 2020;45(Suppl 1):17–27. 32910490 10.1111/jcpt.13230 PMC7540167

[4] Almandoz JP Lingvay I Morales J Campos C. Switching between glucagon-like peptide-1 receptor agonists: rationale and practical guidance. Clin Diabetes. 2020;38:390–402. 33132510 10.2337/cd19-0100 PMC7566932

[5] Leslie RD Pozzilli P Peters AL. Response to the comment on: “Dulaglutide treatment results in effective glycaemic control in latent autoimmune diabetes in adults (LADA): A post-hoc analysis of the AWARD-2, -4 and -5 trials.”. Diabetes Obes Metab. 2018;20:2319–20. 29781106 10.1111/dom.13366

[6] Van J Frias JP Bonora E. Gastrointestinal tolerability of once-weekly dulaglutide 3.0mg and 4.5mg: a post hoc analysis of the incidence and prevalence of nausea, vomiting, and diarrhea in AWARD-11. Diabetes Ther. 2021;12:2783–94. 34514554 10.1007/s13300-021-01140-9 PMC8479017

[7] Cashen K Petersen T. Diabetic ketoacidosis. Pediatr Rev. 2019;40:412–20. 31371634 10.1542/pir.2018-0231

[8] Pozzilli P Pieralice S. Latent autoimmune diabetes in adults: current status and new horizons. Endocrinol Metab (Seoul, Korea). 2018;33:147–59. 10.3803/EnM.2018.33.2.147 PMC6021307 29947172

[9] Nadhem O Nakhla E Smalligan RD. Diabetic ketoacidosis as first presentation of latent autoimmune diabetes in adult. Case Rep Med. 2015;2015:821397. 25834574 10.1155/2015/821397 PMC4365329

[10] Eng C Kramer CK Zinman B Retnakaran R. Glucagon-like peptide-1 receptor agonist and basal insulin combination treatment for the management of type 2 diabetes: a systematic review and meta-analysis. Lancet. 2014;384:2228–34. 25220191 10.1016/S0140-6736(14)61335-0

[11] Varanasi A Bellini N Rawal D. Liraglutide as additional treatment for type 1 diabetes. Eur J Endocrinol. 2011;165:77–84. 21646283 10.1530/EJE-11-0330

[12] Janzen KM Steuber TD Nisly SA. GLP-1 agonists in type 1 diabetes mellitus. Ann Pharmacother. 2016;50:656–65. 27252246 10.1177/1060028016651279

[13] Unger J. Rationale use of GLP-1 receptor agonists in patients with type 1 diabetes. Curr Diab Rep. 2013;13:663–8. 23955813 10.1007/s11892-013-0404-x

[14] Godoy-Matos AF. The role of glucagon on type 2 diabetes at a glance. Diabetol Metab Syndr. 2014;6:91. 25177371 10.1186/1758-5996-6-91 PMC4148933

[15] Gallwitz B. Clinical perspectives on the use of the GIP/GLP-1 receptor agonist tirzepatide for the treatment of type-2 diabetes and obesity. Front Endocrinol (Lausanne). 2022;13:04044. 10.3389/fendo.2022.1004044 PMC9606350 36313764

[16] Ahren B Hirsch IB Pieber TR.; ADJUNCT ONE Investigators. Efficacy and safety of liraglutide added to capped insulin treatment in subjects with type 1 diabetes: the ADJUNCT TWO randomized trial. Diabetes Care. 2016;39:1693–701. 27493132 10.2337/dc16-0690

[17] Mathieu C Zinman B Hemmingsson JU.; ADJUNCT ONE Investigators. Efficacy and safety of liraglutide added to insulin treatment in type 1 diabetes: the ADJUNCT ONE Treat-To-Target randomized trial. Diabetes Care. 2016;39:1702–10. 27506222 10.2337/dc16-0691

[18] Zhang Z Zhang Q Tan Y. GLP-1RAs caused gastrointestinal adverse reactions of drug withdrawal: a system review and network meta-analysis. Front Endocrinol (Lausanne). 2023;14:49328. 10.3389/fendo.2023.1149328 PMC10359616 37484944

[19] Alduraibi RK Alrebdi YM Altowayan YF. Euglycemic diabetic ketoacidosis after the initiation of dulaglutide in patient with type 2 diabetes. Medicine (Baltim). 2023;102:e34027. 10.1097/MD.0000000000034027 PMC10256347 37335652

[20] Okiro JO Mc Hugh C Abdalla A. Is it safe to acutely discontinue insulin therapy in patients with chronic hyperglycaemia starting GLP-1R agonists. BMJ Case Rep. 2017;2017:bcr–2017. 10.1136/bcr-2017-220437 PMC5534999 28710307

